# Solid-Phase Insertion of *N*-mercaptoalkylglycine Residues into Peptides

**DOI:** 10.3390/molecules24234261

**Published:** 2019-11-22

**Authors:** Spyridon Mourtas, Dimitrios Gatos, Kleomenis Barlos

**Affiliations:** 1 Department of Chemistry, University of Patras, 26510 Rio Patras, Greece; s.mourtas@upatras.gr (S.M.); d.gatos@upatras.gr (D.G.); 2 Institute of Chemical Engineering Sciences of the Foundation for Research and Technology Hellas (FORTH/IEC-HT), 26504 Rio Patras, Greece; 3 CBL-Patras, Patras Industrial Area, Block 1, 25018 Patras, Greece

**Keywords:** aminothiols, oxidation, peptoids, solid-phase synthesis, 2-chlorotrityl resin

## Abstract

*N*-mercaptoalkylglycine residues were inserted into peptides by reacting *N*-free amino groups of peptides, which were initially synthesized on 2-chlorotrityl resin (Cltr) using the Fmoc/^t^Bu method, with bromoacetic acid and subsequent nucleophilic replacement of the bromide by reacting with *S*-4-methoxytrityl- (Mmt)/*S*-trityl- (Trt) protected aminothiols. The synthesized thiols containing peptide–peptoid hybrids were cleaved from the resin, either protected by treatment with dichloromethane (DCM)/trifluoroethanol (TFE)/acetic acid (AcOH) (7:2:1), or deprotected (fully or partially) by treatment with trifluoroacetic acid (TFA) solution using triethylsilane (TES) as a scavenger.

## 1. Introduction

**Figure 1:**
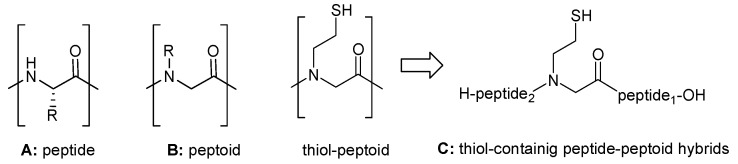
Comparison of a peptide (**A**), a peptoid (**B**), and the newly synthesized thiol-containing peptide–peptoid structure (**C**).

Peptides as pharmaceutical compounds suffer from unfavorable pharmacokinetics such as slow uptake into cells and rapid proteolytic cleavage, among other factors. *N*-alkylation of peptides can overcome these limitations, thus, structures such as *N*-alkylated/methylated peptides [1,2] and *N*-substituted glycine residues (peptoids) [3,4] are of great importance. Oligomers of *N*-substituted glycine residues (peptoids) differ from peptides in that the side chains are connected to amide nitrogen atoms rather than carbon atoms (Figure 1). They are classified as unnatural molecules that confer a high degree of resistance to proteolytic degradation, while the absence of any backbone hydrogen bonding means that peptoids exhibit a high degree of conformational flexibility [5,6], while they also present a broad variety of biological activities [7,8,9,10,11,12,13,14,15,16]. They also present bio-nanotechnological and bio-medicinal interest as novel therapeutics [17,18,19,20], while peptide–peptoid hybrids (peptomers) are also interesting structures which mimic biologically active peptides [21,22,23]. All of the aforementioned points have contributed to peptoids increasingly being investigated in the field of medicinal chemistry as an option to replace peptides in the development of new therapeutics [24].

The solid-phase synthesis (SPS) of peptoids was a major breakthrough because it greatly increased the synthetic efficiency, synthesis yields, and available side chain diversity, thereby dramatically reducing time and costs [3]. Since then, several methods have been used for the solid-phase synthesis (SPS) of peptoids [25,26,27,28] including the use of 2-chlorotrityl resin (Cltr) [29,30].

The thiol group is a structural element which is contained in important biologically active compounds, like peptides, proteins, coenzymes (acetyl coenzyme A), and vitamins (biotin). Examples of FDA-approved drugs include the enzyme inhibitors captopril (which is approved as an Angiotensin-Converting Enzyme (ACE) inhibitor for the treatment of hypertension) [31,32] and thiorphan (the active metabolite of the antidiarrheal racecadotril (acetorphan), which prevents the degradation of endogenous enkephalins by acting as an enkephalinase inhibitor) [33], tiopronin (another approved drug against heavy metal poisoning, liver diseases, or cystenuria), *N*-acetylcysteine (used to treat paracetamol (acetaminophen) overdose and to loosen thick mucus in individuals with cystic fibrosis or chronic obstructive pulmonary disease), and amifostine (a cytoprotective adjuvant used in cancer chemotherapy and radiotherapy) [34], among others.

It is therefore of great interest to develop methods for the solid-phase synthesis (SPS) of thiol-containing peptide–peptoid hybrids in order to mimic cysteine-like structures. For this reason, we prepared *S*-protected aminothiols, which were linear or derived from naturally occurring aminoacids, and applied using solid-phase peptide synthesis (SPPS), which is the most common method of peptide production [35] by applying common methods of peptoid synthesis.

In general, peptoid submonomers are synthesized using a two-step procedure. The first step is an acylation step with bromoacetic acid, using *N*,*N*′-diisopropylcarbodiimide (DIC) as the condensing agent, while the second step is nucleophilic displacement using a monosubstituted amine, resulting in the peptoid submonomer **B** (Figure 1).

By using *S*-4-methoxytrityl- (Mmt)/*S*-trityl- (Trt) protected aminothiols, we envisioned the incorporation of the peptoid submonomer into peptide–peptoid hybrids like molecule **C** (Figure 1). Solid-phase peptide synthesis (SPPS) using Cltr and fluorenylmethoxycarbonyl (Fmoc)/tert-butyl (^t^Bu) was the selected method, which is now the most commonly used methodology for the preparation of peptides [35]. The use of the *S*-Mmt and *S*-Trt protecting groups is of high importance, since *S*-Mmt derivatives can be removed selectively in the presence of ^t^Bu-type protecting groups, thereby enabling selective derivatization via alkylation, pegylation, labeling of the liberated thiol, or synthesis of cystine analogues of peptide–peptoid hybrids after oxidation of the liberated thiols, as examples. Thus, besides the general method for the preparation of the hybrid **C**, we also provide selected examples for the applicability of our method in the synthesis of the protected or deprotected (fully or partially) thiol-containing peptide–peptoid hybrids, and the preparation of inter-disulfide bridged peptide–peptoid structures based on the oxidation of different thiol groups of peptoid submonomers that are present on the peptide chain.

## 2. Results and Discussion

Peptoids are usually synthesized using the submonomer synthetic approach consisting of two steps. The first is an acylation step of the available amine group by bromoacetic acid and diisopropylcarbodiimide (DIC) (which acts as an activator of the bromoacetic acid carboxylic group), whilst the second involves nucleophilic displacement using a monosubstituted amine. This procedure results in peptoid structure **B** (Figure 1). It is of high importance that no racemization was observed by the application of the submonomer method for peptoid synthesis [36,37,38].

**Scheme 1:**
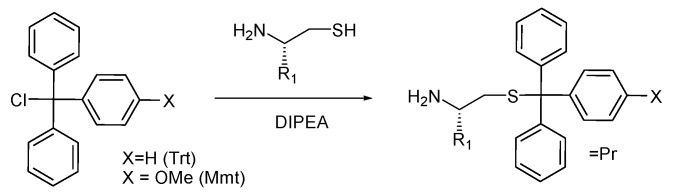
Synthesis of *S*-Mmt/*S*-Trt aminothiols. R_1_ = H or the side chain of naturally occurring aminoacids.

By using *S*-4-methoxytrityl- (Mmt)/*S*-trityl- (Trt) protected aminothiols, we envisioned the solid-phase synthesis of peptoid–peptide hybrids like molecule **C** (Figure 1). For this, we first synthesized *S*-Mmt- and *S*-Trt-protected derivatives of cysteamine or aminothiols derived from optically active aminoacids [39] by their reaction with trityl- or 4-methoxytrityl chloride in the presence of *N*,*N*-diisopropylethylamine (DIPEA) as a base (Scheme 1). The derived *S*-Mmt/Trt-protected aminothiols were applied in the synthesis of thiol-containing peptide–peptoid hybrids (Figure 1) using solid phase peptide synthesis (SPS) and fluorenylmethoxycarbonyl (Fmoc)/tert-butyl (^t^Bu) methodology and 2-chlorotrityl resin (Cltr), which is now the most commonly used methodology for the production of peptides [38].

**Scheme 2:**
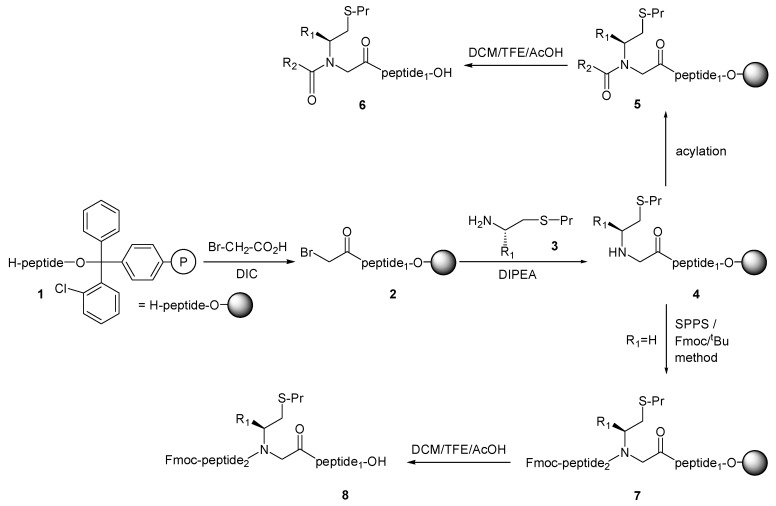
Method for the synthesis of peptide–peptoid hybrids on 2-chlorotrityl resin. Pr = Mmt, Trt; R_1_ = H or amino acid side chain; R_2_ = Ac or Ar.

For this, resin-bound peptide **1** was assembled on 2-chlorotrityl resin [40] (Scheme 2) using *N*-Fmoc-amino acids and 1-hydroxy benzotriazole (HOBt)/DIC for their activation. Then, bromoacetic acid was coupled on the *N*-terminal function of **1** using DIC in *N*-methyl-2-pyrrolidone (NMP). The obtained bromoacetyl resin-bound peptide **2** was further reacted with the aminothiol **3**, which was protected at its thiol function with the acid-sensitive Trt and Mmt groups. In all cases, the nucleophilic replacement of the bromine atom in **2** with the aminothiol **3** was completed within 12 h at room temperature, resulting in the thiol-containing resin-bound peptide **4**.

**Figure 2:**
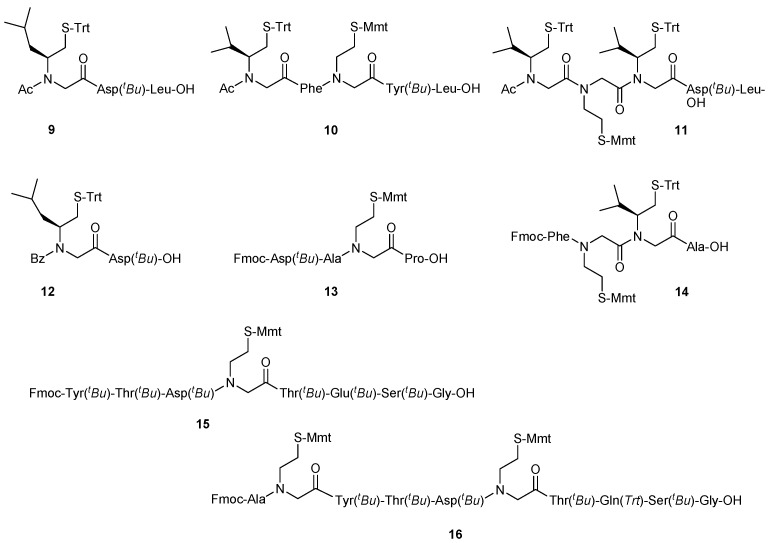
The synthesized *S*-Mmt/*S*-Trt/^t^Bu-protected peptide–peptoid hybrids.

At this step of the proposed synthetic procedure, two options existed. In the first option, acylation of **4** with acetic anhydride (Ac_2_O), acyl (Ac) chlorides, and aroyl (Ar) chlorides in NMP/DIPEA gave **5** within 2 h at room temperature. Treatment of **5** with dichloromethane (DCM)/trifluoroethanol (TFE)/acetic acid (AcOH) (7:2:1) for 15 min at room temperature gave the acylated mercaptoalkyl peptoid **6**. By applying this method, the peptoids **9**–**12** (Figure 2) were prepared and analyzed by LC-MS (Table 1). The second option included the effective acylation by the coupling reaction of **4** with acids such as bromoacetic acid in NMP using DIC as the condensing agent, thereby enabling the insertion of another peptoid submonomer in the peptide chain or the continuation of peptide synthesis. In the latter case, resin-bound aminothiol containing peptide **4** was chain-elongated by using DIC/HOBt or 2-(1H-Benzotriazole-1-yl)-1,1,3,3-tetramethylaminium hexafluorophosphate (HBTU)/DIPEA/HOBt [41,42], but only in the case of using *S*-protected cysteamine as the mercaptoalkyl moiety (R_1_=H). In contrast, the coupling of Fmoc-amino acids with the more steric-hindered aminothiol containing peptide **4** could not be completed under these conditions, regardless of the use of excess amino acid or excess activation reagent. At the end of the synthesis, treatment of **7** with DCM/TFE/AcOH (7:2:1) gave the peptide–peptoid hybrid **8**. By applying this method, the peptoids **13**–**16** (Figure 2) were prepared and analyzed by LC-MS (Table 1). High purities (>88%) were seen using LC-MS analysis for all synthesized derivatives (**9**–**16**), as can be seen by the summarized results in Table 1.

**Scheme 3:**
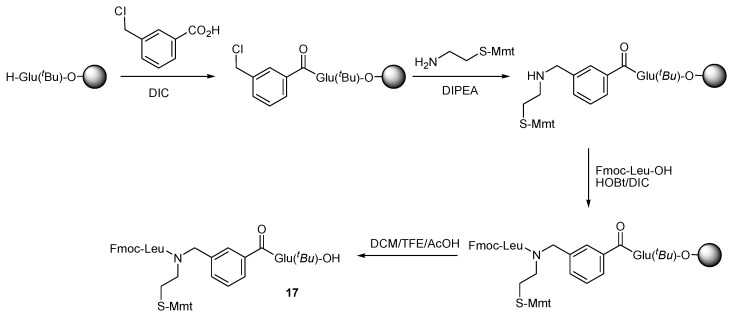
Synthesis of peptide–peptoid hybrid **17** using *m*-chlorobenzoic acid.

Besides bromoacetic acid, coupling with the resin-bound peptide **1** and subsequent replacement of the halogen was also performed using the *m*- and *p*-chloromethylbenzoic acid. In one example of this approach, H-Glu(^t^Bu)-2-Cltr resin reacted with *p*-chloromethylbenzoic acid in the presence of DIC. The derived product was then coupled with Fmoc–Leucine in the presence of HOBt/DIC, and the derived hybrid was cleaved from the resin in its protected form using DCM/TFE/AcOH (7:2:1) to the hybrid **17** (Scheme 3), which was also analyzed by LC-MS (Table 1).

**Scheme 4:**
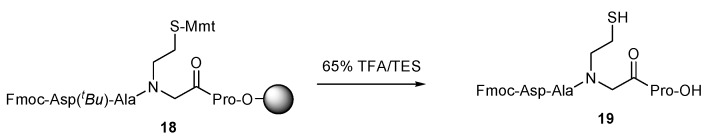
Synthesis of *S*-free/completely unprotected peptide–peptoid **19**.

**Figure 3:**
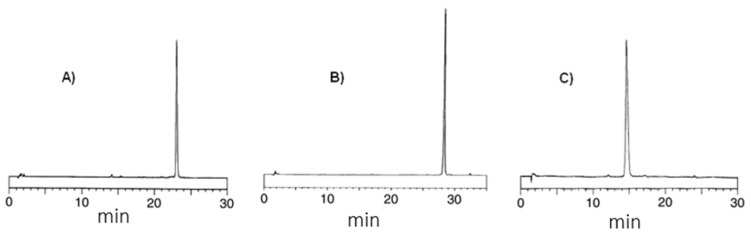
HPLC analysis of peptide–peptoid hybrids: **10** (**A**) (column: Zorbax SB-C18, 3.5 μm; 2.1 × 30 nm; gradient: from 50% to 100% acetonitrile in water within 15 min, 100% acetonitrile within 15 min; flow rate: 0.4 mL/min); **17** (**B**) (column: Lichrospher RP-8 (4 × 150 mm, 5 μm); gradient: from 20% to 100% acetonitrile in water within 30 min; flow rate: 1 mL/min); **19** (**C**) (column: Lichrospher RP-8 (4 × 150 mm, 5 μm); gradient: from 20% to 100% acetonitrile in water within 30 min; flow rate: 1 mL/min). All chromatograms were detected at 265 nm.

As an example of the HPLC analysis chromatograms of the different products **9**–**17**, we present the synthesis of the *S*-Mmt-protected peptide–peptoid hybrids **10** and **17**. These were obtained after treatment of the resin-bound peptide–peptoid hybrids with DCM/TFE/AcOH (7:2:1), which resulted in the cleavage of the *S*-Mmt-protected peptide–peptoid hybrids from the resins (Figure 3A,B). In addition, in order to investigate the effect of different acidic conditions, we treated the resin-bound hybrid **18** with 65% trifluoroacetic acid (TFA)/triethylsilane (TES) (95:5) (Scheme 4). Under these conditions, the hybrid was cleaved from the resin, while the *S*-Mmt and ^t^Bu groups were simultaneously removed to form hybrid **19**. This was prepared in high purity (Figure 3C).

**Scheme 5:**
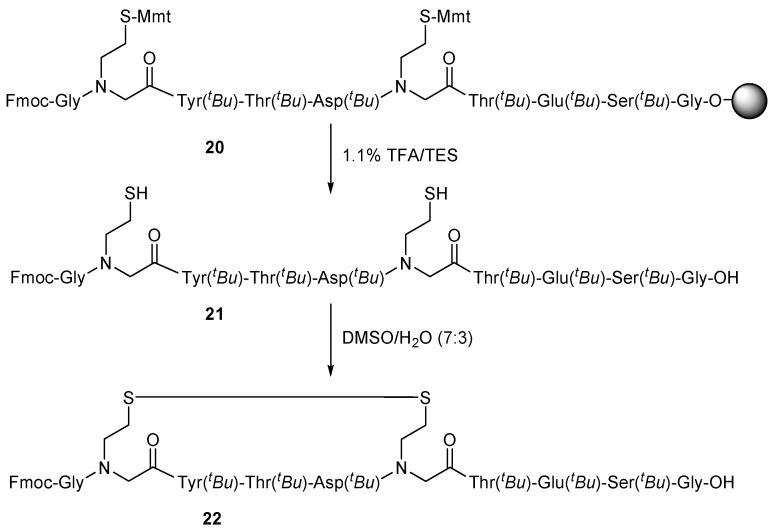
Synthesis of thiol-bridged peptoid–peptide hybrid **22**.

Disulfides in proteins play an important role in the maintenance of biological activity and conformational stability, thus, their formation is often a crucial final stage in peptide synthesis [43]. For this reason, we were also interested in evaluating the applicability of our method in the synthesis of thiol-bridged peptoid–peptide hybrids. As an example, we synthesized the resin-bound peptide-peptoid hybrid **20** (Scheme 5), which contained two *S*-Mmt-protected peptoid submonomers and ^t^Bu-protected amino acids. This was treated with 1.1% TFA in DCM/TES (95:5) for 5 min at room temperature, thereby enabling the cleavage of the hybrid from the resin and selectively removing the *S*-Mmt groups, while the ^t^Bu-groups remained unaffected.

**Figure 4:**
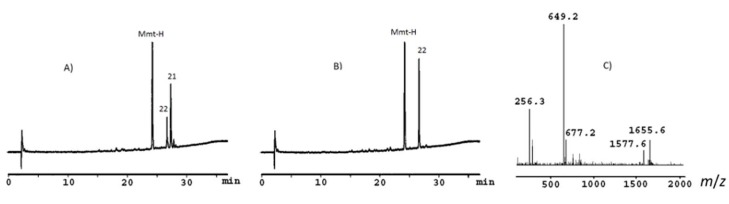
LC-MS analysis of the oxidation of crude hybrid **21** in DMSO, after 12 h (**A**) and 48 h (**B**). Column: Nucleosil C8, 7 μm, 125 × 4 mm; flow rate: 1 mL/min; gradient: from 50 to 100% acetonitrile in water within 30 min; detection at 265 nm. (**C**) ES-MS analysis of the oxidized dimer **22**. [M + Na]^+^ calc: 1655.80; [M + Na]^+^ found: 1655.6.

Via this procedure, the partially ^t^Bu-protected hybrid **21** was obtained and further oxidized using dimethyl sulfoxide (DMSO), which is known as a mild oxidant. The progress of the oxidation was followed by LC-MS (Figure 4). As can be seen, the treatment of the resin **20** with 1.1% TFA in DCM/TES (95:5) gave the *S*-free (^t^Bu-protected) hybrid **21**. HPLC analysis of the crude hybrid showed the existence of Mmt-H (identified by ESI-MS), which was the product of the reaction of TES with Mmt cations formed during cleavage/deprotection. Hybrid **21** was further oxidized to the ^t^Bu-protected peptide–peptoid hybrid **22**. Under these mild conditions, oxidation was completed in 48 h without the formation of any by-products, which was shown by LC-MS analysis (Figure 4B). Moreover, by following the HPLC analysis, we were able to observe the conversion of **21** to **22**. Thus, the oxidation of **21** was completed at about 50% in 12 h (Figure 4A), while after 48 h, **21** was completely oxidized to **22** under the applied conditions (Figure 4B). Both products were identified by MS analysis (see Figure 4C for **22**).

**Scheme 6:**
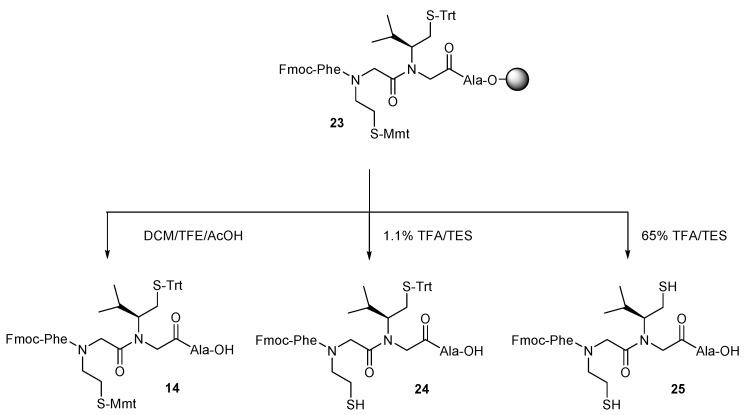
Synthesis of *S*-Mmt/*S*-Trt-protected peptide–peptoid hybrid **14**, partially *S*-free peptide–peptoid hybrid **24**, and *S*-free peptide–peptoid hybrid **25**.

In order to investigate the selective deprotection of the *S*-Mmt over *S*-Trt protecting groups, we studied the gradual removal of the protecting groups of the *S*-Mmt/*S*-Trt type. For this reason we prepared the resin-bound hybrid **23** and initially treated it with 1.1% TFA in DCM/TES (95:5) for 5 min at room temperature (Scheme 6).

**Figure 5:**
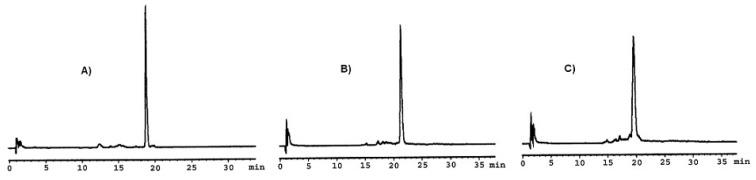
HPLC analysis of hybrids **14** (**A**), **24** (**B**), and **25** (**C**). Column: Zorbax SB-C18, 3.5 μm; 2.1 × 30 nm; gradient: (**A**) from 50% to 100% acetonitrile in water within 15 min, 100% acetonitrile within 15 min; (**B**) from 20% to 100% acetonitrile in water within 30 min; (**C**) from 20% to 100% acetonitrile in water within 30 min; flow rate: 0.4 mL/min; detection at 265 nm.

Under these conditions, the fully *S*-Mmt/*S*-Trt-protected hybrid **14** was synthesized in high selectivity, as shown by HPLC analysis (Figure 5A). In addition, the resin-bound hybrid **23** was also treated with 1.1% TFA in DCM/TES (95:5). Under these conditions, the direct cleavage of the hybrid from the resin and removal of the *S*-Mmt groups enabled the synthesis of the *S*-free/*S*-Trt-protected hybrid **24** (Figure 5B). Finally, the resin-bound hybrid **23** was treated with 65% TFA in DCM/TES (95:5), which resulted in the cleavage of the hybrid from the resin and the simultaneous removal of both *S*-Mmt/*S*-Trt groups to form the fully unprotected hybrid **25** (Figure 5C).

## 3. Materials and Methods 

### 3.1. Materials

All chemicals were purchased from Sigma-Aldrich OM, Athens, Greece, except 2-chlorotrityl polystyrene resin (Cltr) and Fmoc-protected amino acids, which were gifted from CBL Patras S.A. (Industrial area of Patras, Building block 1, GR-25018, Patras, Greece). All chemicals were used without further purification.

### 3.2. Analytical Methods

Thin layer chromatography (TLC) was performed on precoated silica gel 60 F254 plates (Merck, Darmstadt, Germany) and spot detection was carried out by UV light or by charring with a ninhydrin solution. HPLC analysis was performed on a Waters 600E multisolvent delivery system (Milford, MA, USA), combined with Waters 991 photodiode array detector, using the following methods: (A) column: Nucleosil C8, 7 μm, 125 × 4 mm; flow rate: 1 mL/min; gradient: from 50 to 100% acetonitrile in water within 30 min; (B): column: Zorbax SB-C18, 3.5 μm; 2.1 × 30 nm; gradient: from 50% to 100% acetonitrile in water within 15 min, 100% acetonitrile within 15 min or from 20% to 100% acetonitrile in water within 30 min or from 20% to 100% acetonitrile in water within 30 min; flow rate: 0.4 mL/min; (C): Lichrospher RP-8 (4 × 150 mm, 5 μm); gradient: from 20% to 100% acetonitrile in water within 30 min; flow rate: 1 mL/min. All chromatograms were detected at 265 nm. ES-MS spectra were recorded on a Micromass Platform L.C. (Manchester, UK) at 30 V.

### 3.3. Synthetic Procedures

#### 3.3.1. General Protocol for the Synthesis of *S*-trityl (Trt) and *S*-4-methoxytrityl (Mmt) Aminothiols

Cysteamine or aminothiols derived from optically active amino acids (1 mol) [34] were dissolved in the highest possible concentration in dichloromethane (DCM), and diisopropyletheylamine (DIPEA) (2.5 mol) was added. A 1 M solution (with respect to the aminothiol) of trityl chloride (1.2 mol) or 4-methoxytrityl chloride (1.2 mol) in DCM was prepared. This was added dropwise to the stirred aminothiol solution. The completion of the reaction was checked by TLC. The DCM was removed by rotary evaporation and diethylether (DEE) was added to the residual oil. The precipitated solid was filtered, washed with DEE, and dried in vacuo.

#### 3.3.2. Solid-Phase Peptide Assembly, General Protocol

Solid-phase peptide synthesis was carried out manually in plastic syringes equipped with porous polypropylene frits at room temperature using the fluorenylmethoxycarbonyl (Fmoc)/tert-butyl (^t^Bu) strategy [44].

##### Pre-Activation of Fmoc-Amino Acids

Fmoc-amino acid (4.5 mmol) and 1-hydroxybenzotriazole hydrate (HOBt) (5.8 mmol) were dissolved in *N*-methyl-2-pyrrolidone (NMP) (4.5 mL) and cooled to 5 °C. Then, diisopropylcarbodiimide (DIC) (5.0 mmol) was added and the mixture was stirred for 15 min at 5 °C.

##### Coupling

The solution of the pre-activated amino acid was added to the resin-bound amino-acid or peptide or peptide–peptoid hybrid (5.0 g, loading 0.3 mmol/g) and shaken for 3 h at room temperature. A sample was taken to check the reaction completion by Kaiser test. In case of incomplete coupling (positive Kaiser test), recoupling was performed with a fresh solution of activated amino acid (6 mmol).

##### Fmoc-Group Removal and Fmoc-Removal Test

The resin-bound Fmoc-protected amino acids, peptides, or hybrids were treated twice with 25% piperidine in NMP (25 mL each) for 30 min at room temperature. To check the completion of the Fmoc-removal, 25% piperidine in NMP (20 μL) was added to a resin probe (approximately 2 mg) and the mixture was heated for 5 min at 100 °C. From the resulting solution, 10 μL was spotted on a TLC plate and checked under a UV lamp for UV-absorbing material. Alternatively, the solution was injected on HPLC and the Fmoc-material produced and released into solution was quantified at 265 nm. If the Fmoc-removal test remained positive (violet spot under UV) the piperidine treatment was prolonged or repeated until a negative test was achieved. The resin was filtered and washed with NMP (×5), DCM (×3), and DEE (×2), and dried in vacuo; otherwise, it was used directly in the next step.

#### 3.3.3. General Procedure for the Solid-Phase Synthesis of Bromoacylated Peptides

Haloacid (3-fold molar excess over the resin bound peptide) was dissolved in NMP. The solution was cooled on ice and then DIC (equivalent to 3 moles) was added. The reaction mixture was added to the resin-bound peptide and allowed to react for 1–3 h at 25 °C. The resin was filtered and washed with NMP (×5), DCM (×3), and DEE (×2), and dried in vacuo; otherwise, it was used directly in the next step.

#### 3.3.4. General Procedure for Peptoid Formation

A solution of the *S*-Mmt and *S*-Trt aminothiols (2-fold molar excess over the resin bound peptide) in NMP and DIPEA (equivalent to 2.2 moles) was added and the mixture was left to react for 2–12 h at 25 °C. The reaction could be easily followed by TLC and HPLC analysis. Thus 2–3 mg of resin samples were taken and the protected peptides were cleaved from the resin using a 15 min treatment with DCM/trifluoroethanol (TFE)/acetic acid (AcOH) (7:2:1). The resins were then applied to TLC and HPLC analysis. After completion of the synthesis, the resins were extensively washed with NMP (×5), DCM (×3), and DEE (×2), and then dried in vacuo; otherwise, they were used directly in the next step.

The obtained resins were either reacted with acetic anhydride (3 mol), or acyl chlorides (3 mol), or aroyl chlorides (3 mol) in NMP and DIPEA (6 mol) for 1–2 h at 25 °C, or subjected to SPS/SPPS, as previously described. The peptoid formation was easily followed by TLC and HPLC analysis. Thus, 2–3 mg of resin samples were taken and the protected peptides were cleaved from the resin using a 15 min treatment with DCM/TFE/AcOH (7:2:1) and were applied to TLC and HPLC analysis. After completion of the synthesis, the resin was extensively washed with NMP (×5), DCM (×3), and DEE (×2), and then dried in vacuo.

#### 3.3.5. General Procedure for the Cleavage of the Peptide–Peptoid Hybrids from the Resin

Resin (0.5 g) was treated with 5 mL of a mixture of DCM/TFE/AcOH (7:2:1) for 15 min at 25 °C, and washed with DCM (3 × 5 mL). The combined filtrates were concentrated by rotary evaporation. The fully protected crude peptides (*S*-Mmt/*S*-Trt/^t^Bu-protected) were precipitated by the addition of DEE, filtered, washed with DEE, and dried in vacuo. Treatment with 1.1% trifluoroacetic acid (TFA) in DCM/triethylsilane (TES) (95:5) gave the *S*-free (*S*-Trt/^t^Bu-protected) hybrids (by removal of the *S*-Mmt protecting group), while treatment with 65% TFA in DCM/TES (95:5) gave the completely unprotected peptides (by removal of the *S*-Mmt/*S*-Trt/^t^Bu protecting groups).

#### 3.3.6. General Procedure for the Oxidation of Mercaptoalkyl Peptoid Side Chains

100 mg of resin was treated with 1 mL of 1.1% TFA in DCM/TES (95:5) for 10 min at 25 °C. This treatment resulted in the cleavage of the protected peptide–peptoid hybrid from the resin and the simultaneous removal of the *S*-Mmt groups. In the case of the *S*-Trt protecting groups, a solution of 65% TFA in DCM/TES (95:5) for 1 h at 25 °C was necessary for the complete removal of the *S*-Trt and ^t^Bu-type protecting groups. The resin was filtered off and 0.2 mL methanol (MeOH) was added to the filtrates. The mixture was left for 5 min at room temperature and then evaporated. Dimethylsulfoxide (DMSO)/water (H_2_O) (7:3) was added to the oily residue and the mixture was left at room temperature for 48 h. The progress of the oxidation was followed by HPLC analysis.

## 4. Conclusions

Peptoids are very important peptide-mimicking compounds, since they can resist proteolytic degradation, thereby retaining a high degree of conformational flexibility. Thiols are very important chemical groups found in drugs and peptides with very important biological activities. Thus, we synthesized thiol-containing peptide–peptoid hybrids using standard methods of solid-phase synthesis (SPS). For this, suitably protected aminothiols (*S*-4-methoxytrityl (Mmt)/*S*-trityl (Trt)), linear and derived from amino acids, were used, and peptide synthesis was performed on 2-chlorotrityl resin (Cltr) using the fluorenylmethoxycarbonyl (Fmoc)/tert-butyl (^t^Bu) strategy. This approach allowed for the selective deprotection of the side groups, giving many possibilities regarding peptide synthesis, including (a) the preparation of peptide–peptoid hybrids with selected thiol-free peptoid moieties (which have the ability to act as new nucleophiles) and (b) the synthesis of peptide–peptoid hybrids with inter- and intra-thiol-containing bridges.

## Figures and Tables

**Table 1:** Retention time (t_R_), yield of the crude product (%), HPLC purity of the crude products (%), *m*/*z* (calculated and found) of the peptide–peptoid hybrids **9**–**17**.

a/a	t_R_	Crude Yield	HPLC Purity ^c^	*m*/*z*	
	(min)	(%)	(%)	Calculated	Found
**9**	26.7 ^b^	92	91	760.4 ^d^	760.6 ^d^
**10**	23 ^a^	94	93	1330.63 ^d^	665.9 ^e^
**11**	34 ^b^	92	89	1536.71 ^d^	768.9 ^a^
**12**	27.2 ^b^	89	88	709.33 ^d^	709.3 ^d^
**13**	32 ^b^	95	93	969.41 ^d^	969.4 ^d^
**14**	18.8 ^a^	93	90	1249.52 ^d^	625.4 ^e^
**15**	19.3 ^a^	93	89	1719.89 ^d^	855.0 ^e^
**16**	26.9 ^a^	90	92	2365.13 ^d^	-
**17**	28.3 ^b^	96	92	1004.45 ^d^	1004.4 ^d^

^a^ Column: Zorbax SB-C18, 3.5 μm; 2.1 × 30 nm; gradient: from 50% to 100% acetonitrile in water within 15 min, 100% acetonitrile within 15 min; flow rate: 0.4 mL/min. ^b^ Column: Lichrospher RP-8, 5 μm; 4 × 150 mm; gradient: from 20% to 100% acetonitrile in water within 30 min; flow rate: 1 mL/min. ^c^ HPLC purity determined at 265 nm. ^d^ [M + H]^+^. ^e^ [M + 2H]^+2^.
